# Too risky, too large, too late, or too mild—Reasons for not treating ischemic stroke patients and the related outcomes

**DOI:** 10.3389/fneur.2022.1098779

**Published:** 2022-12-22

**Authors:** Anne Brink Behrndtz, Andreas Gammelgaard Damsbo, Rolf Ankerlund Blauenfeldt, Grethe Andersen, Lasse Ole Speiser, Claus Ziegler Simonsen

**Affiliations:** ^1^Department of Neurology, Aarhus University Hospital, Aarhus, Denmark; ^2^Department of Clinical Medicine, Aarhus University, Aarhus, Denmark; ^3^Department of Neuroradiology, Aarhus University Hospital, Aarhus, Denmark

**Keywords:** mild symptoms, stroke, thrombolytic therapy, no treatment, outcomes

## Abstract

**Background:**

Despite effective treatments, many patients are still not offered reperfusion therapy for acute ischemic stroke.

**Methods:**

We present a single-center observational study on acute ischemic stroke patients, who presented as candidates for reperfusion therapy but were deemed ineligible after work-up. Reasons for non-treatment were obtained by studying patient files and subsequently grouped into “too risky” (e.g., anticoagulant use, comorbidities), “too large” (large infarct), “too late” (late presentation of stroke and wake-up strokes), or “too mild” (clinically mild/remitting symptoms). Modified Rankin scale (mRS) score was prospectively collected in all patients by a structured telephone interview. All non-treated patients with a National Institute of Health Stroke Scale (NIHSS) score of 0–5 were compared with a similar cohort that was treated.

**Results:**

Of 529 patients with acute ischemic stroke arriving as reperfusion therapy candidates, 198 (37.4%) were not treated. The majority (42%) were not treated due to admission outside the treatment window (too late) and 24% had absolute contraindications (too risky). Only 8% was excluded because their infarct was too large [median Alberta Stroke Program Early CT score 3 (2–4)]. In the “too mild” group (14%) the percentage of patients not being independent at 90 days was 30%. The adjusted odds ratio for a better outcome (lower mRS) among treated patients with NIHSS 0–5 compared with non-treated was 1.93 (95% confidence interval 1.15–3.23).

**Conclusion:**

Presenting outside the treatment window is still the most common reason for not receiving therapy. Our study suggests a benefit of thrombolysis for patients with mild symptoms.

## Introduction

Treatment of acute ischemic stroke (AIS) improves outcomes and is proven to be more effective the earlier the treatment is initiated (1). Patient eligibility for intravenous thrombolysis (IVT) has now expanded with a potential increase in the number of acute admissions. The WAKE-UP trial provided the possibility of treating patients presenting with unknown onset using Magnetic Resonance Imaging (MRI) (2). The EXTEND trial proved the benefit of treating within a 9-h window using perfusion imaging (3) and DAWN and DEFUSE-3 have shown the efficacy of endovascular therapy (EVT) up to 24 h after onset in patients selected with advanced imaging (4, 5). In addition, it is now possible to evaluate plasma levels of direct oral anticoagulants and to treat if the levels are low (6). Previous studies on patient and system-related factors for not receiving IVT or EVT have suggested late arrival as the most common reason for not offering treatment (7). The PRISMS trial suggested no benefit of IVT in “non-disabling” strokes (8).

To explore how new guidelines have been taken into daily practice, we aimed to collect the arguments from patient files to reveal all possible arguments for not treating with IVT and EVT. We included consecutive patients presenting with AIS who were deemed relevant for reperfusion therapy evaluation in prehospital telephone triage, and we examined their outcomes. In addition, we wanted to compare outcomes for patients with mild strokes to a cohort of similarly treated patients from the same center.

## Methods

We retrospectively investigated reasons for not offering acute reperfusion treatment on all patients evaluated and diagnosed with AIS in 2018 at our tertiary stroke center. Results from WAKE-UP, DAWN, and DEFUSE-3, (2, 4, 9) had been implemented into clinical practice at the end of 2017.

Referral was performed by either a resident or a stroke neurologist, while decisions on treatment always relied on the stroke neurologist. Prior to the call, emergency medical service (EMS) had performed a prehospital stroke score and obtained the patient history (10). Patients were evaluated as candidates for IVT if they had symptoms of AIS, were independent in activities of daily living, and were last seen well < 4.5-h ago or if they had unknown onset and were last seen well >4.5 h ago. They were evaluated as candidates for EVT if they had severe neurological deficits, were last seen well < 24 h ago, and were independently living. Admission was decided by a teleconference between the on-call neurologist at the nearest stroke unit and the EMS.

Default stroke imaging was MRI, including diffusion weighted imaging (DWI), fluid-attenuated inverse recovery (FLAIR), and T2^*^-weighted imaging. Time-of-flight angiography was performed if the National Institute of Health Stroke Scale (NIHSS) was 6 or above or if a large vessel occlusion (LVO) was suspected (clinically or radiologically), and perfusion imaging could be made on request.

### Data collection

Data on comorbidity, imaging, clinical characteristics, and outcome on both treated and non-treated patients were prospectively collected in the local database for the Danish Stroke Registry (11). Outcome was measured by modified Rankin Scale (mRS) at 90 days and was performed by telephone interview based on the simplified mRS questionnaire (12). The patient was considered independent if mRS was 0-2 and mRS 0-1 was considered an excellent outcome. Data were analyzed as part of a quality project and were thus exempt from ethical approval.

**Figure 1:**
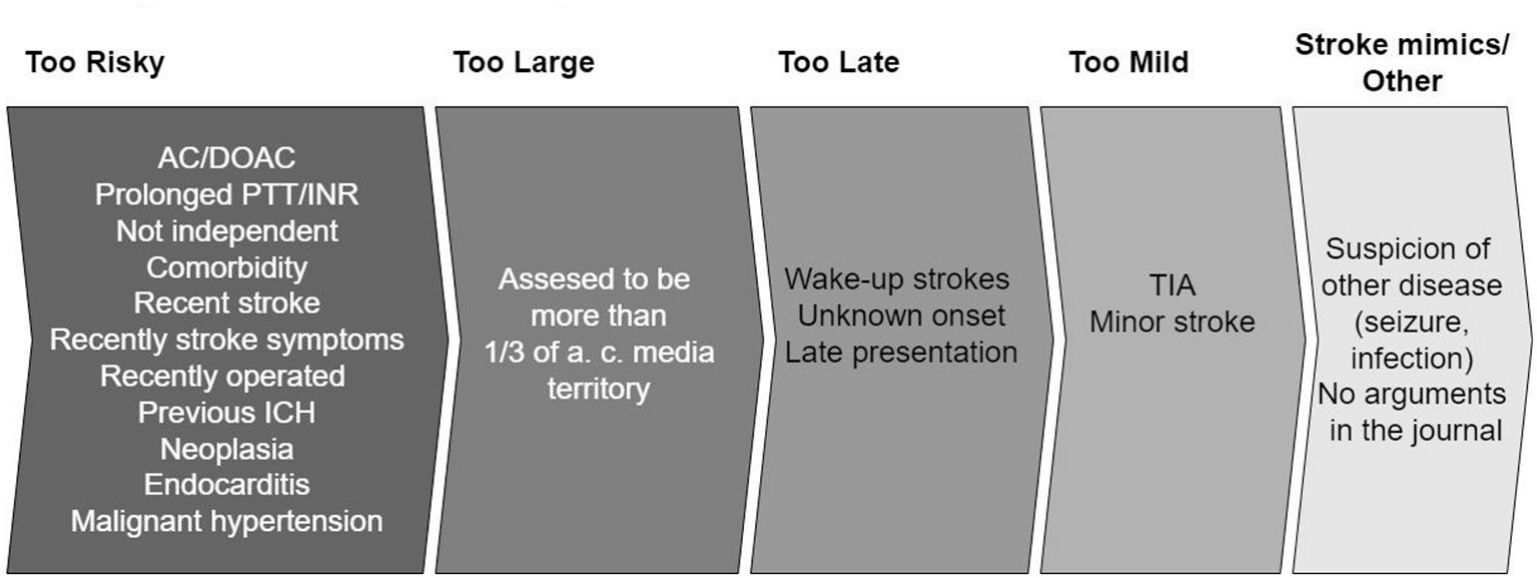
Arguments grouped into five categories. When there is more than one argument, the most important is prioritized in the following order: “Too risky,” “to large,” “too late,” “too mild,” “stroke mimics/other.” A more detailed version of the arguments exists in Appendix Table 1. AC, anticoagulants; DOAC, direct acting oral anticoagulants; PTT, partial thromboplastin time; INR, internationalized normalized ratio; ICH, intracranial hemorrhage; TIA, transitory ischemic attack.

If the patients did not receive IVT or EVT, their charts were read by two independent neurologists to establish the argument(s) not to treat. In cases of disagreements, a consensus was obtained between the assessors. We registered written arguments against treating with IVT or EVT, predefined by the current guidelines (13). The arguments were based on history, prescriptions, examination, biochemistry, and imaging. The aspect score was examined by a doctor in neuroradiology afterward. Arguments were grouped into five categories, see Figure 1. If the patient had one argument in the “too risky” group, this took precedence. Next, the argument “too large” was prioritized. The rest was put in the remaining groups by the same principle in the following order: “too late” and “too mild.” This method was used to simplify the most important arguments and a group of stroke experts made a consensus on the order of “most important” arguments.

Patients with arguments based on imaging were allocated into the four groups in the following way: microbleeds at T2^*^ weighted sequences and recent infarctions in “too risky,” patients referred as EVT candidates (because of severe symptoms) and where acute imaging showed open vessels were put in “too large,” unknown onset with FLAIR positive infarct in “too late” and “no visible infarction on MRI” in “too mild.” The “stroke mimics/other” group was comprised of a patient with unknown reasons for not treating and patients that initially receive another diagnosis (seizure, infection) but later were proven to have a stroke.

Study data were prospectively collected and managed using REDCap electronic data capture tools hosted at Aarhus University Hospital (14). Data can be shared on reasonable request.

### Statistics

Baseline characteristics were compared between treated and non-treated patients using the *t-*test and χ^2^-test, where appropriate.

The distribution of primary rejection arguments is visualized with a pie chart. The distribution of 90 days mRS scores for patients stratified by primary rejection argument is visualized in a stacked bar chart.

We performed both bivariate and multivariate ordinal logistic regression to evaluate the association between 90 days mRS score (ordinal scale) and NIHSS score of five or less. In the multivariate analysis, we included age and NIHSS score as a continuous variable, and the following binary variables (yes/no): female sex, smoking, prior transitory ischemic attack, prior stroke, comorbidities (atrial fibrillation, hypertension, diabetes) and pre-stroke mRS.

Odds ratios are reported with 95 % confidence intervals (CI). The brant-Wald test was used to confirm that the regression assumption of proportional odds holds. All analyses were performed using R 4.1 (R-foundation, Vienna, Austria) (15).

## Results

**Figure 2:**
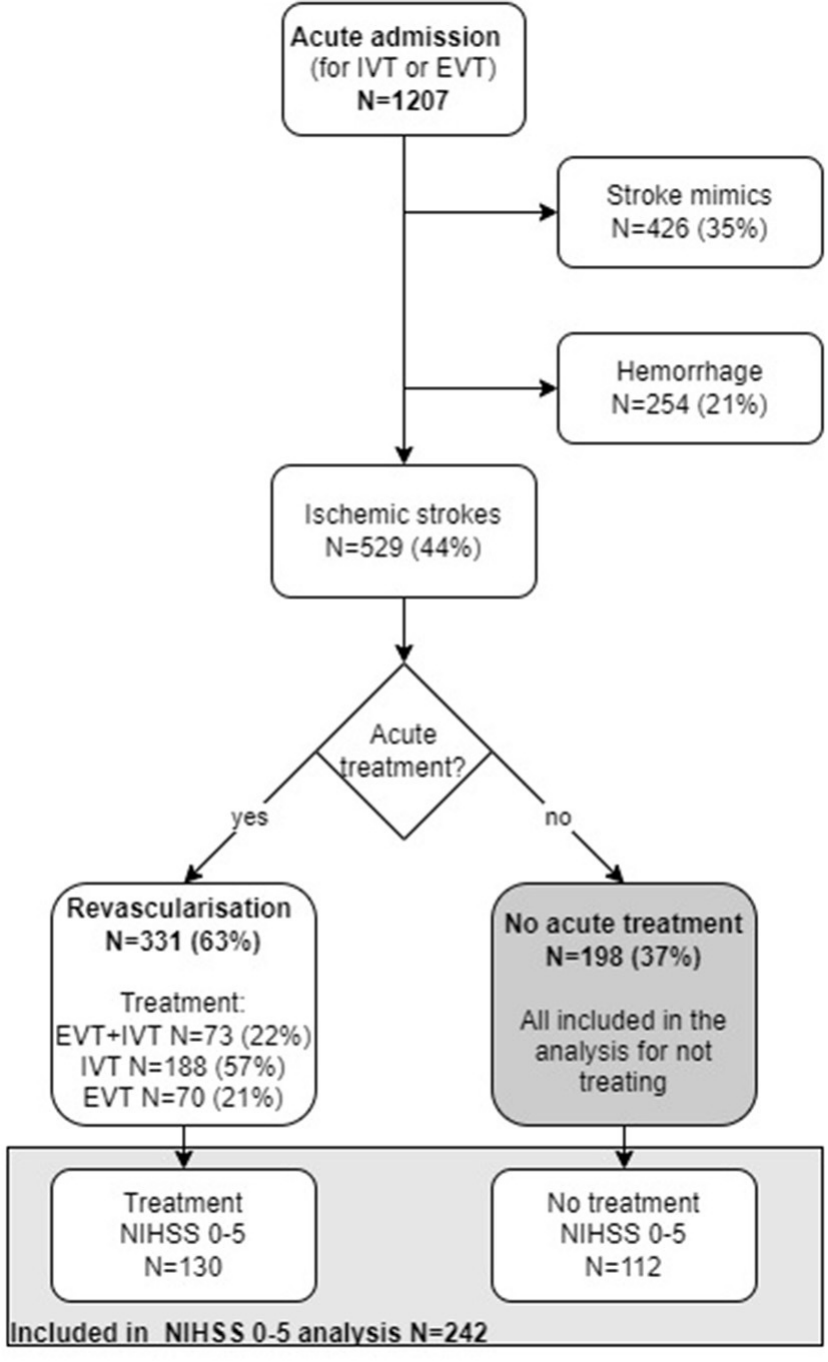
Flow chart illustrating number of acute admissions (N). IVT, Intravenous thrombolysis; EVT, endovascular treatment; NIHSS, National institute of health stroke scale.

In the period Jan 1st, 2018 to Dec 31st, 2018, 1,207 patients were admitted with a putative stroke. Of these, 529 (44%) had an ischemic stroke, 254 (21%) had an intracerebral hemorrhage, and 426 (35%) had a stroke mimic. Of patients with ischemic stroke, 37% (198 out of 529) were not treated with acute reperfusion therapy (Figure 2).

Of the 529 AIS patients, 13 were lost to follow-up for mRS at 90 days and 10 of them were in the not-treated group. In the not-treated population, 90% (*n* = 179) were examined with MRI and in the treated population, 83% (*n* = 274) were examined with MRI (*p* = 0.02). Median onset to imaging time was 268 min (IQR;122–633 min) in the not treated group and 110 min (IQR; 78–178 min) in the treated group.

At admission, 19 patients (10%) of the non-treated population were referred for only EVT and 61 (31%) were referred to as wake-up strokes. The prevalence of each argument is listed in Table 1.

**Table 1:** Prevalence of each argument used in the files.

**Prevalence of arguments in the 198 not treated patients**			
**History**		**Symptoms**	
Unknown onset	22 (11%)	Transitory ischemic attack	11 (5.6%)
Out of IVT window	26 (13%)	Few symptoms	35 (18%)
Recent stroke symptoms	5 (2.5%)	Prolonged appt/INR	11 (5.6%)
Not independently living	6 (3.0%)	Raised DOAK value	1 (0.5%)
AC/DOAK	19 (9.6%)	High blood pressure	2 (1.0%)
Recent bleeding	0 (0%)	Endocarditis	1 (0.5%)
Recent CNS-surgery	2 (1.0%)	**Findings on imaging**	
Recent head trauma	0 (0%)	No visible infarction	9 (4.5%)
Recent large surgery/trauma	1 (0.5%)	Infarction too large	21 (11%)
Recent smaller surgery	2 (1.0%)	Too many microbleeds	6 (3.0%)
Allergy to thrombolysis	0 (0%)	Too flair positive	90 (45%)
Recent Stroke	1 (0.5%)	Other younger infarct	6 (3.0%)
Earlier intracranial hemorrhage	2 (1.0%)	Hemorrhagic transformation	5 (2.5%)
Neoplasia	4 (2.0%)	MRI not possible (wakeup)	6 (3.0%)
Liver disease	0 (0%)	No mismatch (LVO 24 h window)	2 (1.0%)
Comorbidity	4 (2.0%)	Cerebral vascular malformations	0 (0%)
Thrombocytopenia	0 (0%)	Aortic aneurysm	0 (0%)
Other	6 (3.0%)	Other imaging	3 (1.5%)
		Aortic dissection	0 (0%)
		Open vessels (EVT-candidates)	17 (8.6%)

DOAC, Dual anticoagulants; MRI, Magnetic resonance Imaging; LVO, Large Vessel Occlusion.

In the non-treated compared to the treated group, there was a significantly higher prevalence of diabetes (19 vs. 11%), atrial fibrillation (25 vs. 21%), and hypertension (60 vs. 51%) and a lower stroke severity score (NIHSS 4 vs. 8). The age was also higher (76 vs. 75 years, respectively) and there were significantly fewer with premorbid mRS of 0-2 (Table 2). The baseline pattern was generally the same for the mild stroke population (NIHSS 0-5) but in this subgroup occurrence of atrial fibrillation did not differ and facial palsy was not overrepresented in the treated group as in the total population (66 vs. 56%) (Appendix Table 3).

**Table 2:** Baseline characteristics of all treated and not treated patients.

**Baseline characteristics**							
	**Treatment**	**No treatment**	**Too risky**	**Too large**	**Too late**	**Too mild**	**Other**
	**N** = **331**	**N** = **198**	**N** = **51**	**N** = **20**	**N** = **91**	**N** = **33**	**N** = **3**
Age^*^	75 (65, 82)	76 (68, 85)	82 (73, 87)	86 (72, 89)	75 (68, 82)	70 (63, 78)	91 (90, 94)
Female	118 (36%)	81 (41%)	23 (45%)	9 (45%)	32 (35%)	16 (48%)	1 (33%)
Atrial fibrillation^*^	69 (21%)	50 (25%)	24 (47%)	11 (55%)	11 (12%)	3 (9.1%)	1 (33%)
Diabetes^*^	36 (11%)	37 (19%)	11 (22%)	4 (20%)	14 (15%)	7 (21%)	1 (33%)
Hypertension^*^	169 (51%)	118 (60%)	38 (75%)	12 (60%)	46 (51%)	19 (58%)	3 (100%)
Previous AIS	13 (3.9%)	9 (4.5%)	6 (12%)	0 (0%)	2 (2.2%)	1 (3.0%)	0 (0%)
Previous TIA	35 (11%)	18 (9.1%)	7 (14%)	1 (5.0%)	7 (7.7%)	3 (9.1%)	0 (0%)
Smoker	86 (26%)	54 (27%)	9 (18%)	3 (15%)	29 (32%)	13 (39%)	0 (0%)
Previous AMI	37 (11%)	22 (11%)	5 (9.8%)	3 (15%)	8 (8.8%)	6 (18%)	0 (0%)
Peripheral artery disease	18 (5.4%)	14 (7.1%)	4 (7.8%)	4 (20%)	4 (4.4%)	2 (6.1%)	0 (0%)
Living alone	78 (39%)	89 (27%)	22 (43%)	13 (65%)	29 (32%)	12 (36%)	2 (67%)
Premorbid mRS 0-2^*^	310 (94%)	158 (86%)	37 (76%)	16 (89%)	72 (89%)	31 (97%)	2 (67%)
NIHSS^*^	8 (3, 17)	4 (2, 10)	6 (3, 14)	22 (14, 24)	4 (2, 7)	1 (0, 2)	10 (10, 11)
Consciousness^*^	122 (37%)	58 (30%)	19 (39%)	15 (75%)	23 (25%)	0 (0%)	1 (50%)
Eye-palsy/visual loss^*^	153 (46%)	56 (29%)	16 (33%)	15 (75%)	23 (25%)	2 (6.2%)	0 (0%)
Facial palsy^*^	219 (66%)	109 (56%)	31 (63%)	18 (90%)	50 (55%)	9 (28%)	1 (50%)
Motor loss^*^	246 (74%)	111 (57%)	34 (69%)	19 (95%)	50 (55%)	6 (19%)	2 (100%)
Ataxia^*^	55 (17%)	32 (16%)	12 (24%)	0 (0%)	15 (16%)	4 (12%)	1 (50%)
Sensory loss^*^	194 (59%)	79 (41%)	19 (39%)	17 (85%)	36 (40%)	7 (22%)	0 (0%)
Aphasia/Dysarthria^*^	245 (74%)	122 (63%)	33 (67%)	20 (100%)	59 (65%)	8 (25%)	2 (100%)
Inattention^*^	90 (27%)	23 (12%)	5 (10%)	8 (40%)	7 (7.7%)	2 (6.2%)	1 (50%)

Median (IQR); n (%).

* Difference between treatment and no treatment is significant p < 0.05.

AIS, Acute ischemic stroke; TIA, transitory ischemic attack; AMI, acute myocardial infarction; mRs, Modified Rankin scale score; NIHSS, National institutional of health Stroke Scale.

**Figure 3:**
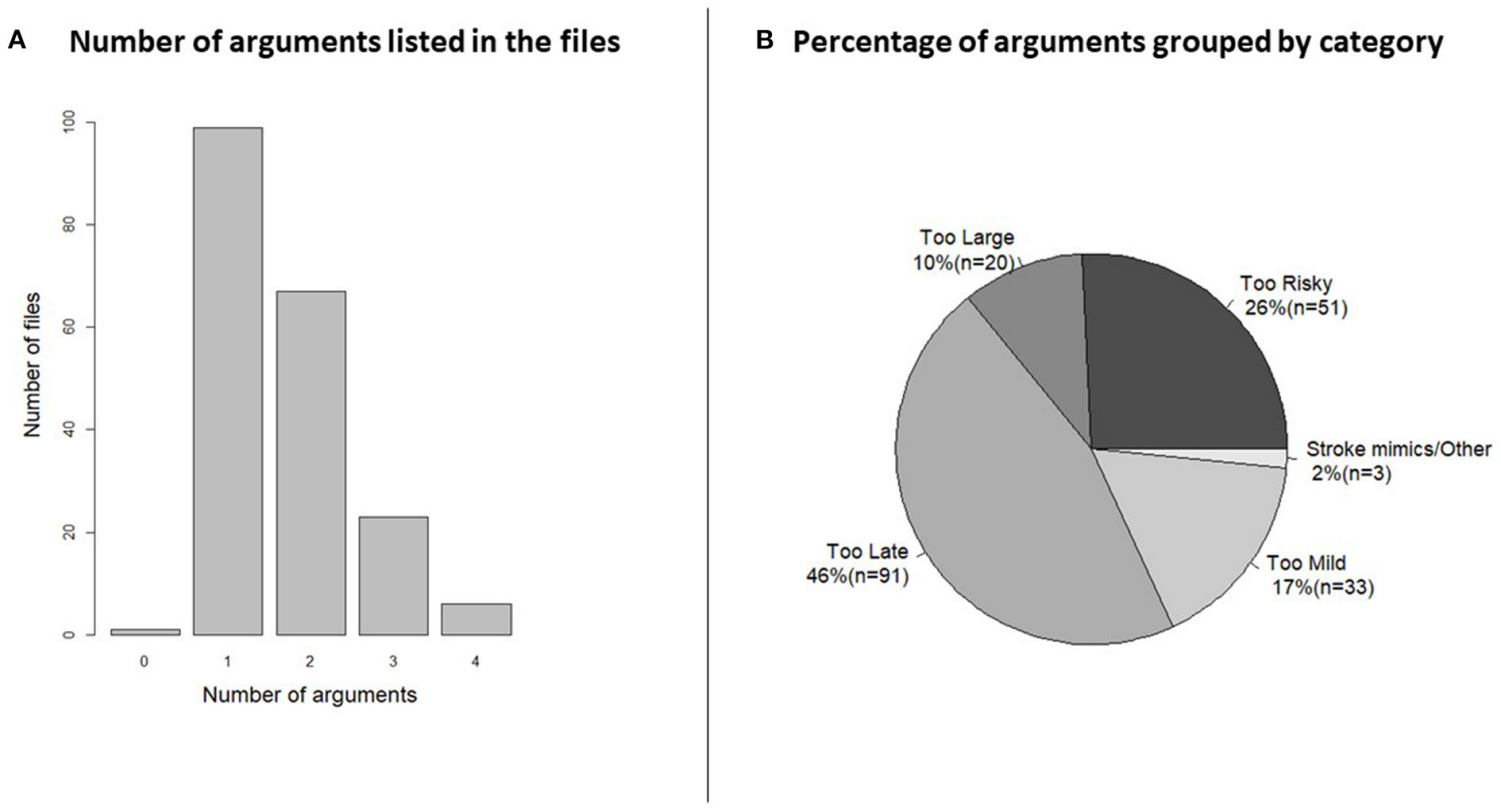
**(A)** Histogram of the number of arguments shows how the half of the patient files had just one argument for not treating but the other half had more than one. Categorization of the arguments was necessary for visualization. **(B)** The pie chart shows the percentage and number (n) of patients in each category. AC, anticoagulants; DOAC, direct acting oral anticoagulants; PTT, partial thromboplastin time; INR, internationalized normalized ratio; ICH, intracranial hemorrhage; TIA, transitory ischemic attack; CVM, cerebrovascular malformation; LVO, large vessel occlusion; IVT, intravenous thrombolysis; EVT, endovascular treatment; CNS, central nervous system.

Of the 198 patients, 99 patients were withheld therapy by one argument, in 67 cases two arguments were used, in 23 cases three arguments were used and in six cases the stroke physician used four arguments for withholding therapy (Figure 3). In one case, no argument was registered. Overall, 30% of the arguments were based on the history, 19% were based on clinical examination of the patient and 50% of the argument were related to imaging, and 1% was unknown.

When arguments were grouped by the most important argument (Figure 3) 91 (46%) patients arrived outside the treatment window (“too late”), where 43 patients (47%) were referred initially as wake-up patients and 48 (53%) were after admission deemed outside the window of treatment. IVT or EVT was considered “too risky” in 51 patients (26%) and of those 22 patients were rejected because of anticoagulation treatment. In 20 patients (10%) the infarct was deemed “too large,” median Alberta Stroke Program Early CT score in this group was 3 (interquartile range: 2–4). Mild symptoms “too mild” were used as the main arguments in 33 (17%) cases.

**Figure 4:**
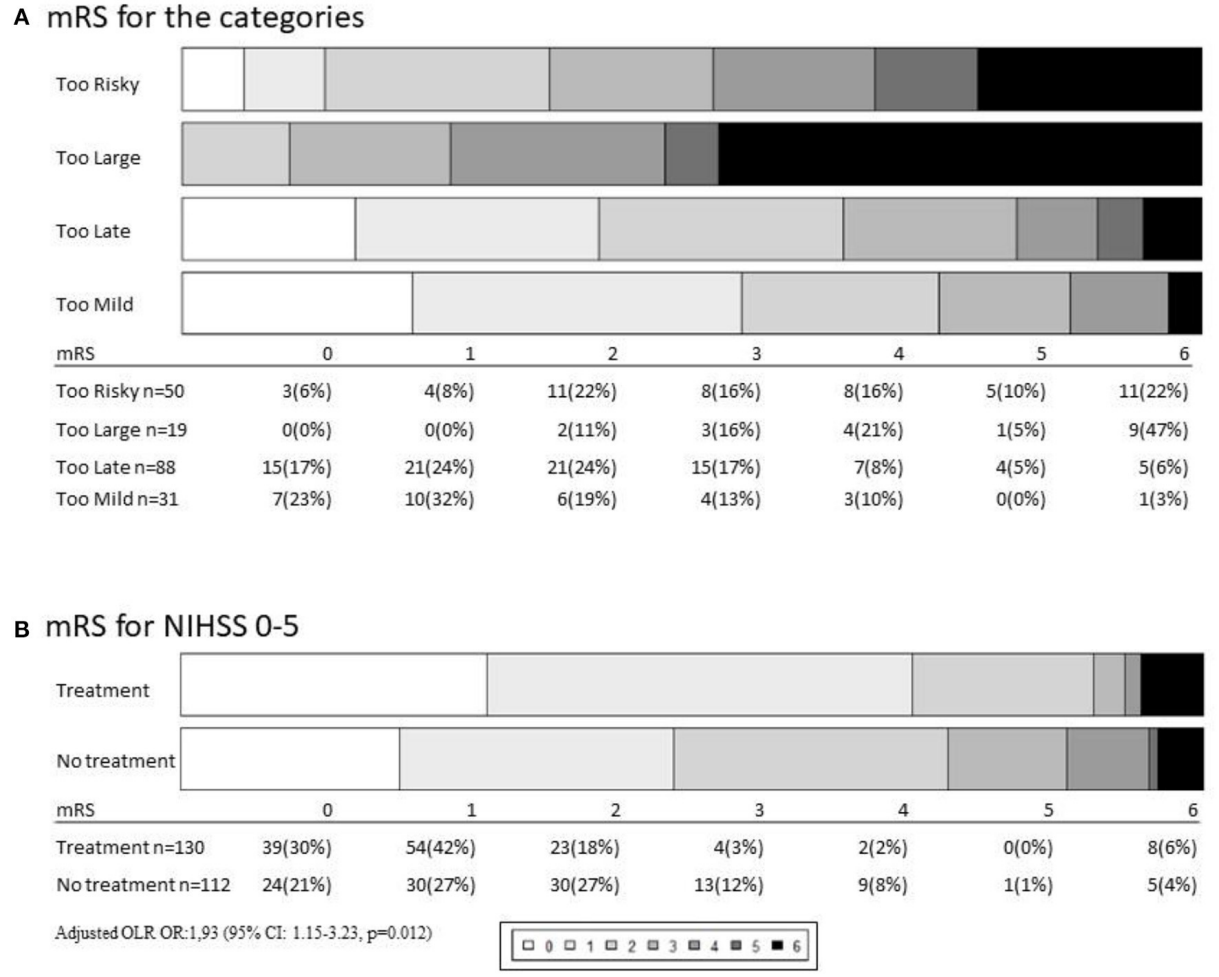
**(A)** Outcomes depending on category measured by 90 days modified Rankin Scale score (mRS). Of 198 not-treated patients the mRS 6 patients were lost to follow up. **(B)** In the National institute of health stroke scale (NIHSS) 0–5 analysis 130 did get treatment with intravenous thrombolysis (IVT) and/or endovascular treatment (EVT) and 112 were not treated.

Functional outcome in the four groups at 90 days is visualized in Figure 4. Of the patients with “too large” infarctions, 10% were independent after 90 days and 45% had died. In the “too risky” group, 35% were independent after 90 days. Of the 22 patients not treated because of oral anticoagulant use, 45% were independent after 90 days. The percentage of patients in the “too late” group that were independent at 90 days was 63% (Figure 4).

In patients in the category “too mild,” where treatment was deferred based on too few symptoms, independency was found in 70% of the cases.

The fact that 30% of the mild strokes were not independent after 30 days led to an analysis of all strokes in our cohort with NIHSS of 0–5 (*n* = 242) where 112 patients were not-treated and 130 patients had treatment primarily with IVT (9 was treated with EVT). In this analysis, the percentage of patients not being independent (mRS 3-6) was 25% (*n* = 28) in the not-treated population whereas 11% (*n* = 14) in the treated group was found not independent at 90 days. When adjusted for age, sex, pre-stroke mRS, NIHSS, and pre-stroke comorbidity, we found an odds ratio of 1.93 [95% confidence interval (CI): 1.15–3.23, *p* = 0.012] of achieving a better outcome if treating mild strokes NIHSS 0-5 (Figure 4, Appendix Table 2). Arguments against treatment are listed in Appendix Figure 1.

In 39 (20%) of the non-treated patients, the main argument was solely based on imaging. In this group, 20 patients were not treated due to a large infarct, 5 patients because no infarct was seen on MRI, 7 because the infarct was FLAIR positive, 2 because of finding another subacute lesion (1 also with microbleeds), 1 with too many microbleeds, and 4 were EVT-only candidates and had no visible vessel occlusion. In two cases, another disease than stroke was suspected in the acute setting but the diagnosis was changed afterward.

## Discussion

In this single-center, retrospective analysis, we found that the most common reason for not treating AIS patients with reperfusion therapy was a late presentation (46%). This was followed by factors rendering treatment too risky (26%), too mild symptoms (17%), and finally a too large infarct (10%). Only 70% of patients with mild symptoms were living independently after 90 days. We found a potential benefit of IVT with an odd ratio for the shift to a lower mRS score of 1.93 (*p* = 0.012) when comparing the group with mild symptoms that were treated with the non-treated group.

Comparing our study to other studies with predefined templates, this study revealed some arguments not defined as absolute contraindications by the guidelines, i.e., imaging arguments and mild strokes (7). In addition, nine out of ten in our study had a brain MRI in the acute phase which increases the validity of the stroke diagnosis (16).

In this study arriving too late is the most common cause of not receiving treatment. In a similar study from 2001, the amount of “too late” patients was 72 % (17). Our finding (46 %) is probably explained by the reduced patient and prehospital delay and extended windows for treatments (18). When excluding the patients referred as wake-up candidates, the patients arriving too late are reduced to 43 patients of all not-treated patients (22%) which indicates that both prehospital triage and delay for stroke patients have been optimized, increasing the chances of getting IVT (19).

The treatment window introduced by WAKE-UP increases admission load and in our study 48 patients were admitted with wake-up strokes and not treated with IVT. This is ~4% of all the acute admissions (*n* = 1,207) that year. In the WAKE-UP trial, 37% of the screened patients received IVT. Compared with our general treatment rate (331/1,207 = 27.4%) this probably justifies the evaluation of WAKE-UP patients.

When comparing outcomes for the “too late” patients in our study with the WAKE-UP trial, the proportion of not-treated patients who had mRS 0-2 after 90 days was 62%, which corresponds well to the proportion of patients randomized to placebo in the WAKE-UP trial (65%). An important note is that the latter group all had FLAIR negative infarctions. This indicates that being FLAIR positive is not in itself a predictor of outcome, but just an indicator of infarction age.

The “too risky” group in our cohort mainly consists of patients taking direct oral anticoagulants (DOACs) (43%). Some patients have been shown to be eligible for IVT when carefully selected depending on drug-specific assays or the opportunity of reversal therapies (20, 21). Discussion remains regarding DOAC treatment and treating patients with IVT where there is the uncertainty of the last DOAC dose, risk of clotting, and last known well.

Mild strokes are maybe not that mild. A large “get with the guidelines paper” found that 28.5% of patients with mild stroke were not able to ambulate at discharge (22). We found that 30% of the “too little” group was not independently living at 90 days. When comparing all patients with mild strokes, the chance of a better outcome was doubled (OR 1.93) in the case of treatment. This suggests an effect of IVT in this population with mild strokes (8). A limitation to this statement is that this study is retrospective and the patients selected for treatment were deemed as good candidates potentially resulting in confounding by indication. Other confounders we did not adjust for, such as stroke site and presence of arterial occlusion, could potentially also affect the results.

The PRISMS trial was terminated early because of futility. It did not find IVT beneficial for patients with non-disabling stroke (23). The MaRISS trial is prospective and observational, and this would hopefully help further clarify if IVT is beneficial in mild strokes and in patients with rapid improvement of symptoms regardless of NIHSS (24). In this context it should be noted that the vast majority in both our groups of treated or non-treated patients had MRI (88% in the treated group and 90% in the not-treated) which enrich our population with “true” stroke patients. PRISMS was primarily a CT trial. We speculate that the benefit of treatment exists when the diagnosis is more certain (DWI positivity) and reversely that the prognosis is better in the case of DWI-negative stroke (25). Other groups have found MRI to be safe for selecting patients with mild stroke for IVT (26).

It is a limitation of the study that it is made in a single center and with a retrospective design. Due to the design, the clinicians were not attentive to registering the arguments systematically in the files. The study is also rather small, but this might be compensated by the data quality. Another limitation is that the trial has been carried out before the publication of the EXTEND trial (3) and meta-analysis (27), and in a setting where a 4, 5–9 h window for IVT is used, there may be a higher proportion of treated patients in the late time window. But in this setting, a higher proportion of acute admitted who is not treated in the late window could also appear.

## Conclusion

Most AIS patients unable to receive treatment were referred with unknown onset or outside the treatment window. One-fourth of the AIS patients did not receive treatment because the symptoms were considered too mild. This study suggests a benefit of IVT in mild strokes.

## Data availability statement

The raw data supporting the conclusions of this article will be made available by the authors, without undue reservation.

## Ethics statement

Ethical review and approval was not required for the study on human participants in accordance with the local legislation and institutional requirements. Written informed consent from the patients/participants or patients/participants' legal guardian/next of kin was not required to participate in this study in accordance with the national legislation and the institutional requirements.

## Author contributions

AB gathered all information from files, made the first analysis, and made the first draft of the article. AD helped with statistics and writing. RB helped with idea development and writing. GA helped collecting files and writing. LS helped viewing scans and files. CS helped with writing and concept development. All authors contributed to the writing of the manuscript.

## References

[1] Lees KR Bluhmki E von Kummer R Brott TG Toni D Grotta JC. Time to treatment with intravenous alteplase and outcome in stroke : an updated pooled analysis of ECASS, ATLANTIS, NINDS, and EPITHET trials. Lancet. (2010) 375:1695–703. 10.1016/S0140-6736(10)60491-6 20472172

[2] Thomalla G Simonsen CZ Boutitie F Andersen G Berthezene Y Cheng B. MRI-guided thrombolysis for stroke with unknown time of onset. N Engl J Med. (2018) 379:611–22. 10.1056/NEJMoa1804355 29766770

[3] Ma H Campbell BCV Parsons MW Churilov L Levi CR Hsu C. Thrombolysis guided by perfusion imaging up to 9 hours after onset of stroke. N Engl J Med. (2019) 380:1795–803. 10.1056/NEJMoa1813046 31067369

[4] Nogueira RG Jadhav AP Haussen DC Bonafe A Budzik RF Bhuva P. Thrombectomy 6 to 24 hours after stroke with a mismatch between deficit and infarct. N Engl J Med. (2017) 378:11–21. 10.1056/NEJMoa1706442 29129157

[5] Jadhav AP Desai SM Kenmuir CL Rocha M Starr MT Molyneaux BJ. Eligibility for endovascular trial enrollment in the 6-to 24-hour time window analysis of a single comprehensive stroke center. Stroke. (2018) 49:1015–7. 10.1161/STROKEAHA.117.020273 29581344

[6] Shahjouei S Tsivgoulis G Goyal N Sadighi A Mowla A Wang M. Safety of intravenous thrombolysis among patients taking direct oral anticoagulants: a systematic review and meta-analysis. Stroke. (2020) 51:533–41. 10.1161/STROKEAHA.119.026426 31884908

[7] Messé SR Reeves MJ Smith EE Saver JL Fonarow GC Schwamm LH. Why are acute ischemic stroke patients not receiving IV tPA? results from a national registry. Neurology. (2016) 87:1565–74. 10.1212/WNL.0000000000003198 27629092 PMC5067546

[8] Khatri P Kleindorfer DO Devlin T Jr RNS Starr M Mejilla J. Effect of alteplase vs aspirin on functional outcome for patients with acute ischemic stroke and minor nondisabling neurologic deficits the PRISMS Randomized Clinical Trial. JAMA. (2018) 320:156–66. 10.1001/jama.2018.8496 29998337 PMC6583516

[9] Albers GW Marks MP Kemp S Christensen S Tsai JP Ortega-Gutierrez S. Thrombectomy for stroke at 6 to 16 hours with selection by perfusion imaging. N Engl J Med. (2018) 378:708–18. 10.1056/NEJMoa1713973 29364767 PMC6590673

[10] Gude MF Blauenfeldt RA Behrndtz AB Nielsen CN Speiser L Simonsen CZ. The prehospital stroke score and telephone conference: a prospective validation. Acta Neurol Scand. (2022) 145:541–50. 10.1111/ane.13580 35023151

[11] Paaske Johnsen S Ingeman A Hundborg HH Schaarup SZ Gyllenborg J Johnsen SP. The Danish Stroke Registry. Clin Epidemiol. (2016) 8:697–702 10.2147/CLEP.S103662 27843349 PMC5098511

[12] Bruno A Akinwuntan AE Lin C Close B Davis K Baute V. Simplified modified rankin scale questionnaire reproducibility over the telephone and validation with quality of life. Stroke. (2011) 42:2276–9. 10.1161/STROKEAHA.111.613273 21680905

[13] Powers WJ Rabinstein AA Ackerson T Adeoye OM Bambakidis NC Becker K. 2018 Guidelines for the early management of patients with acute ischemic stroke: a guideline for healthcare professionals from the American Heart Association/American Stroke Association. Stroke. (2018) 49:e46–110. 10.1161/STR.0000000000000158 29367334

[14] Harris PA Taylor R Thielke R Payne J Gonzalez N Conde JG. Research electronic data capture (REDCap)-A metadata-driven methodology and workflow process for providing translational research informatics support. J Biomed Inform. (2009) 42:377–81. 10.1016/j.jbi.2008.08.010 18929686 PMC2700030

[15] RStudio. Index @ http://Www.Rstudio.Com. (2018). Available online at: http://www.rstudio.com/ (accessed December 12, 2022).

[16] Chalela JA Kidwell CS Nentwich LM Luby M Butman JA Demchuk AM. Magnetic resonance imaging and computed tomography in emergency assessment of patients with suspected acute stroke: a prospective comparison. Lancet. (2007) 369:293–8. 10.1016/S0140-6736(07)60151-2 17258669 PMC1859855

[17] Barber PA Uk M Zhang J Demchuk AM Hill MD Buchan AM. Why are stroke patients excluded from TPA therapy? an analysis of patient eligibility. Neurology. (2001) 56:1015–20. 10.1212/WNL.56.8.1015 11320171

[18] Lees KR Emberson J Blackwell L Bluhmki E Davis SM Donnan GA. Effects of alteplase for acute stroke on the distribution of functional outcomes a pooled analysis of 9 trials. Stroke. (2016) 47:2373–9. 10.1161/STROKEAHA.116.013644 27507856 PMC5024752

[19] Prabhakaran S O'Neill K Stein-Spencer L Walter J Alberts MJ. Prehospital triage to primary stroke centers and rate of stroke thrombolysis. JAMA Neurol. (2013) 70:1126–32. 10.1001/jamaneurol.2013.293 23817961

[20] Seiffge DJ Wilson D Wu TY. Administering thrombolysis for acute ischemic stroke in patients taking direct oral anticoagulants: To treat or how to treat. JAMA Neurol. (2021) 78:515–6. 10.1001/jamaneurol.2021.0287 33720313

[21] Czap AL Grotta JC Stroke HM. Complexities of reperfusion therapy in patients with ischemic stroke pretreated with direct oral anticoagulants to treat or not, and how? JAMA Neurol. (2021) 78:517–8. 10.1001/jamaneurol.2021.0290 33720276

[22] Smith EE Fonarow GC Reeves MJ Cox M Olson DM Hernandez AF. Outcomes in mild or rapidly improving stroke not treated with intravenous recombinant tissue-type plasminogen activator: findings from get with the guidelines-stroke. Stroke. (2011) 42:3110–5. 10.1161/STROKEAHA.111.613208 21903949

[23] Benoit JL Khatri P Adeoye OM Broderick JP McMullan JT Scheitz JF. Prehospital triage of acute ischemic stroke patients to an intravenous tPA-ready versus endovascular-ready hospital: a decision analysis. Prehospital Emerg Care. (2018) 22:722–33. 10.1080/10903127.2018.1465500 29847193

[24] Romano JG Gardener H Campo-Bustillo I Khan Y Riley N Tai S. The mild and rapidly improving stroke study (MaRISS): rationale and design. Int J Stroke. (2019) 14:983–6. 10.1177/1747493019873595 31496438

[25] Simonsen CZ Madsen MH Schmitz ML Mikkelsen IK Fisher M Andersen G. Sensitivity of diffusion-and perfusion-weighted imaging for diagnosing acute ischemic stroke is 97. %. Stroke. (2015) 46:98–101. 10.1161/STROKEAHA.114.007107 25388415

[26] Majidi S Luby M Lynch JK. MRI-based thrombolytic therapy in patients with acute ischemic stroke presenting with a low NIHSS. Neurology. (2019) 93:E1507–13. 10.1212/WNL.0000000000008312 31519779 PMC6815207

[27] Campbell BCV Ma H Ringleb PA Parsons MW Churilov L Bendszus M. Extending thrombolysis to 4·5–9 h and wake-up stroke using perfusion imaging: a systematic review and meta-analysis of individual patient data. Lancet. (2019) 394:139–47. 10.1016/S0140-6736(19)31053-0 31128925

