# Supplementary material for: Too risky, too large, too late, or too mild—Reasons for not treating ischemic stroke patients and the related outcomes
Source: Front Neurol. 2022 Dec 22;13:1098779. doi: 10.3389/fneur.2022.1098779 (PMC9815765; doi:10.3389/fneur.2022.1098779)
Supplement: Supplementary file 1 [file Data_Sheet_1.pdf]

Supplementary Appendix

This appendix has been provided to give readers additional information about their work.

**History:**	"Unknown onset",<br>"Out of window",<br>"Recent stroke symptoms"<br>,"not independent",<br>"AC/NOAC",<br>"Recent bleeding",<br>"Recent CNS-surg",<br>"recent head trauma",<br>"CVM",<br>"recent large op/trauma",<br>"recent small op",<br>"allergy",<br>"recent stroke",<br>"earlier ICH", "Neoplasia",<br>"Liver disease",<br>"comorbidity",<br>"Thrombopenia",<br>"other in hist"
**Examination:**	"TIA”<br>"Few symptoms”<br>"prolonged PTT/INR”<br>"raised DOAC value"<br>"high blood pressure”<br>"endocarditis”<br>”other findings”
**Imaging**:	"no visible infarction",<br>"infarction to large",<br>"to many microbleeds",<br>"to flair positive",<br>"Recent other infarction",<br>"Hemorrhagic transformation",<br>"no MRI (wake-up)",<br>"No mismatch(extended window)",<br>"CVM",<br>"Aortic aneurysm",<br>"Aortic dissection",<br>"other imaging",<br>"open vessels(EVT only)",<br>"susp seizure",<br>"susp CNS inf",<br>"other"

Table 1. List of all arguments registered in redcap database

	Bivariate ordinal logistic regression				Multivariate ordinal logistic regression		
Characteristic	N	OR[1]	95% CI[1]	p-value	OR[1]	95% CI[1]	p-value
**Treatment**	242	2.15	1.35, 3.44	**0.001**	1.93	1.15, 3.23	**0.012**
**Age (each 1 year)**	242	1.03	1.01, 1.05	**<0.001**	1.02	1.0, 1.04	0.13
**Female sex**	242	0.81	0.50, 1.31	0.4	0.83	0.50, 1.39	0.5
**AFIB**	242	1.00	0.52, 1.90	>0.9	0.68	0.34, 1.36	0.3
**Diabetes**	242	2.85	1.48, 5.51	**0.002**	1.77	0.87, 3.63	0.12
**Hypertension**	242	2.26	1.43, 3.61	**<0.001**	1.55	0.93, 2.59	0.091
**Prior TIA**	242	0.78	0.36, 1.65	0.5	0.96	0.44, 2.06	>0.9
**Prior stroke**	242	1.67	0.55, 5.06	0.4	1.05	0.33, 3.29	>0.9
**Current smoker**	242	0.86	0.52, 1.43	0.6	0.91	0.52, 1.58	0.7
**NIHSS (each 1 point)**	242	1.29	1.11, 1.51	**0.001**	1.25	1.06, 1.49	**0.008**
**premorbid mrs**	231	0.08	0.02, 0.35	**0.001**	0.17	0.03, 0.79	**0.025**

[1]OR = Odds Ratio, CI = Confidence Interval

Table 2. Shift analysis showing the unadjusted (bivariate) and adjusted (multivariate) ordinal logistic regression models of the NIHSS 0-5 group and the covariates the multivariate analysis is adjusted for. We present how the variables influence the shift in mRS.

Baseline characteristics for nihss 0-5	No treatment	Treatment	
Characteristic	N = 115[1]	N = 133[1]	P-value[2]
Age	75 (68, 81)	73 (64, 80)	0.087
Female	34 (30%)	50 (38%)	0.2
Atrialfibrillation	19 (17%)	16 (12%)	0.3
Diabetes	22 (19%)	14 (11%)	0.055
Hypertension	70 (61%)	59 (44%)	0.009
Prev.AIS	6 (5.2%)	6 (4.5%)	0.8
Prev.TIA	13 (11%)	11 (8.3%)	0.4
Smoker	34 (30%)	32 (24%)	0.3
Previous AMI	12 (10%)	11 (8.3%)	0.6
Periferal artery disease	8 (7.0%)	4 (3.0%)	0.15
Living alone	35 (30%)	39 (29%)	0.8
Premorbid mRS	100 (96%)	132 (99%)	0.2
NIHSS			
0	12 (10%)	5 (3.8%)	0.038
1	28 (24%)	29 (22%)	0.6
2	24 (21%)	26 (20%)	0.8
3	26 (23%)	24 (18%)	0.4
4	16 (14%)	28 (21%)	0.14
5	9 (7.8%)	21 (16%)	0.055
Consciousness	7 (6.1%)	10 (7.5%)	0.7
Eye-palsy/visual loss	6 (5.2%)	10 (7.5%)	0.5
Facial palsy	49 (43%)	54 (41%)	0.7
Motor loss	35 (30%)	61 (46%)	0.013
Ataxia	23 (20%)	36 (27%)	0.2
Sensory loss	30 (26%)	41 (31%)	0.4
Aphasia/Dysarthiria	51 (44%)	65 (49%)	0.5
Inattention	5 (4.3%)	4 (3.0%)	0.7

[1] Median (IQR); n (%);

[2] Wilcoxon rank sum test; Pearson's Chi-squared test; Fisher's exact test

Table 3. Baseline characteristics for NIHSS 0-5 group.

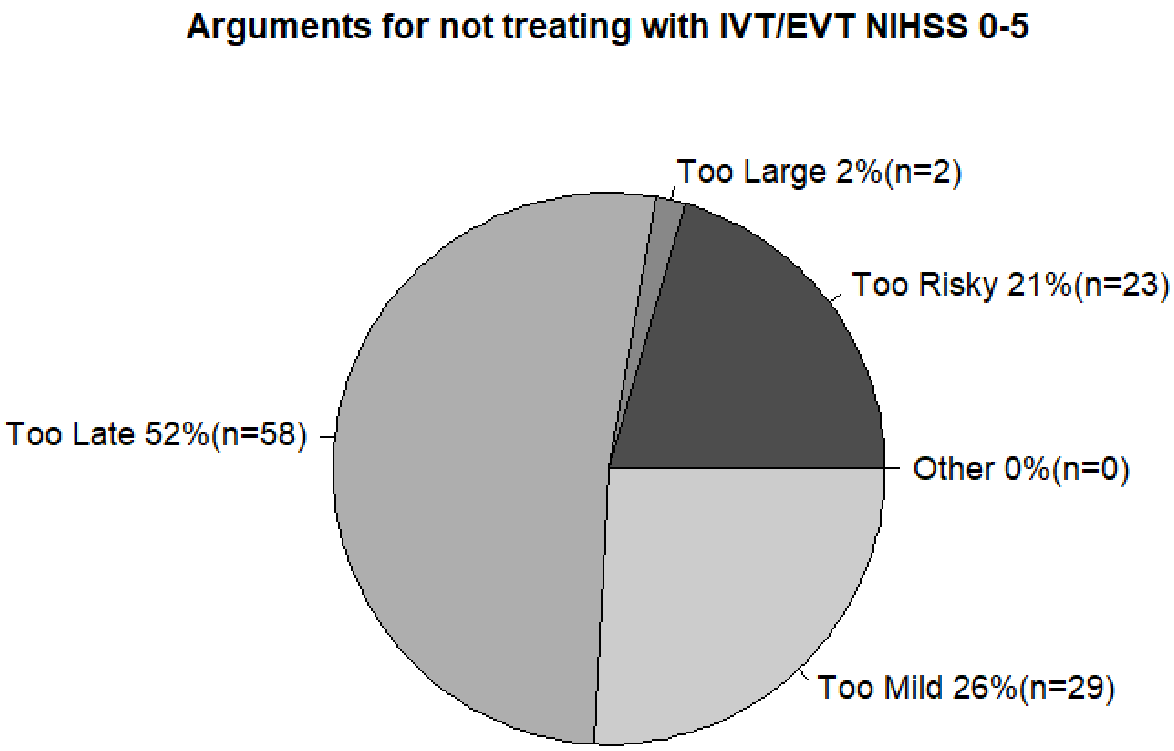

Figure1. Arguments for not treating in the group of NIHSS 0-5.
